# Denoising EEG Signals for Real-World BCI Applications Using GANs

**DOI:** 10.3389/fnrgo.2021.805573

**Published:** 2022-01-13

**Authors:** Eoin Brophy, Peter Redmond, Andrew Fleury, Maarten De Vos, Geraldine Boylan, Tomás Ward

**Affiliations:** ^1^School of Computing, Dublin City University, Dublin, Ireland; ^2^Infant Research Centre, University College Cork, Cork, Ireland; ^3^Insight SFI Research Centre for Data Analytics, Dublin City University, Dublin, Ireland; ^4^Transpoco Telematics, Dublin City University Alpha Innovation Campus, Dublin, Ireland; ^5^Department of Electrical Engineering, KU Leuven, Leuven, Belgium; ^6^Department of Development and Regeneration, KU Leuven, Leuven, Belgium

**Keywords:** EEG, denoising, GAN, BCI, time series

## Abstract

As a measure of the brain's electrical activity, electroencephalography (EEG) is the primary signal of interest for brain-computer-interfaces (BCI). BCIs offer a communication pathway between a brain and an external device, translating thought into action with suitable processing. EEG data is the most common signal source for such technologies. However, artefacts induced in BCIs in the real-world context can severely degrade their performance relative to their in-laboratory performance. In most cases, the recorded signals are so heavily corrupted by noise that they are unusable and restrict BCI's broader applicability. To realise the use of portable BCIs capable of high-quality performance in a real-world setting, we use Generative Adversarial Networks (GANs) that can adopt both supervised and unsupervised learning approaches. Although our approach is supervised, the same model can be used for unsupervised tasks such as data augmentation/imputation in the low resource setting. Exploiting recent advancements in Generative Adversarial Networks (GAN), we construct a pipeline capable of denoising artefacts from EEG time series data. In the case of denoising data, it maps noisy EEG signals to clean EEG signals, given the nature of the respective artefact. We demonstrate the capability of our network on a toy dataset and a benchmark EEG dataset developed explicitly for deep learning denoising techniques. Our datasets consist of an artificially added mains noise (50/60 Hz) artefact dataset and an open-source EEG benchmark dataset with two artificially added artefacts. Artificially inducing myogenic and ocular artefacts for the benchmark dataset allows us to present qualitative and quantitative evidence of the GANs denoising capabilities and rank it among the current gold standard deep learning EEG denoising techniques. We show the power spectral density (PSD), signal-to-noise ratio (SNR), and other classical time series similarity measures for quantitative metrics and compare our model to those previously used in the literature. To our knowledge, this framework is the first example of a GAN capable of EEG artefact removal and generalisable to more than one artefact type. Our model has provided a competitive performance in advancing the state-of-the-art deep learning EEG denoising techniques. Furthermore, given the integration of AI into wearable technology, our method would allow for portable EEG devices with less noisy and more stable brain signals.

## 1. Introduction

**Figure 1:**
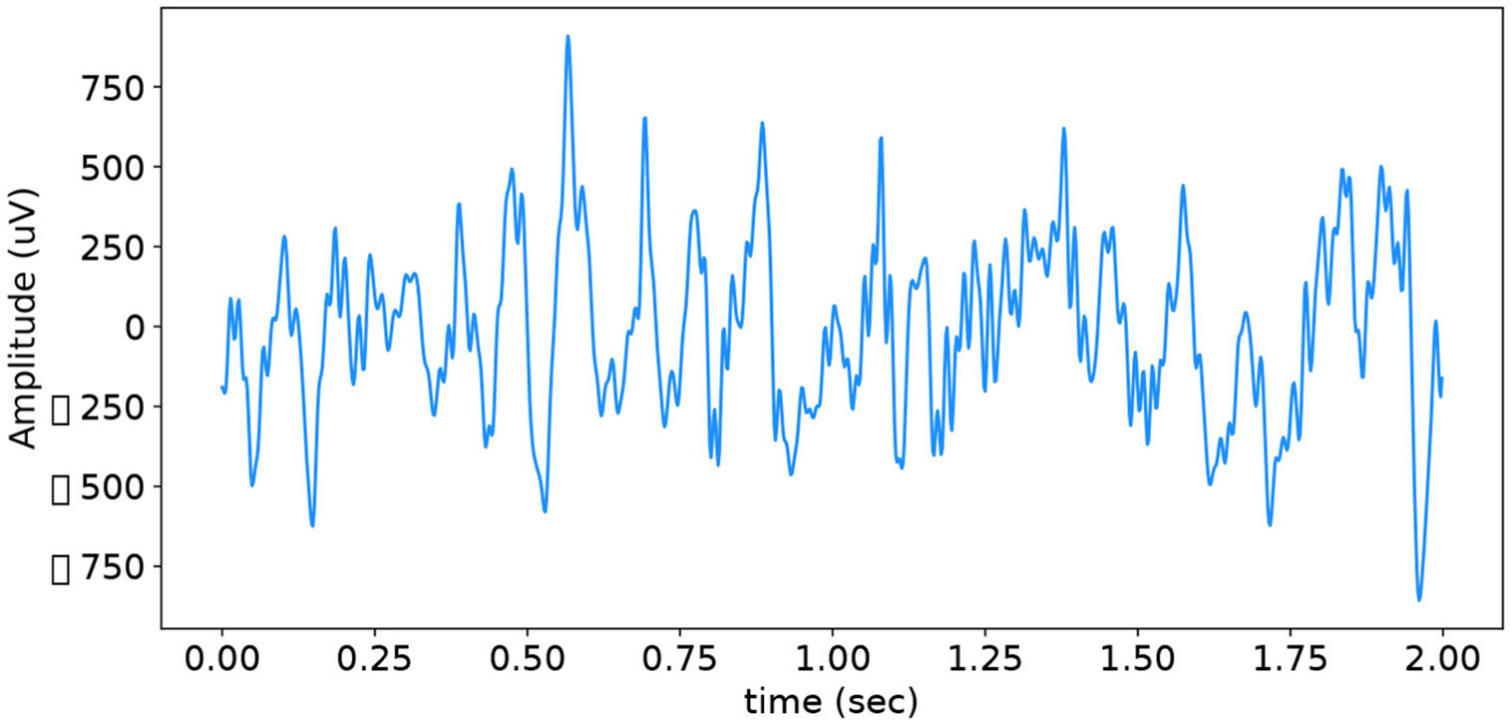
Typical example of clean EEG.

Electroencephalography (EEG) is a method of measuring the electrical activity of the brain. It is a non-invasive procedure that obtains measurements via several electrodes placed on the scalp of the patient. See Figure 1 for an example of clean EEG. EEG has become an essential tool for practitioners in diagnosing abnormal brain activity and neurological conditions such as epilepsy. A recurring issue with EEG readings is that they can be heavily corrupted with artefacts induced from muscle movements, electrical interference or loose electrodes, to name a few. These artefacts make classification and, consequently, diagnosis of neurological conditions a bottleneck. As a result, denoising EEG has become an extensive area of research in the biomedical signal processing domain (Anderer et al., 1999; Jiang et al., 2019).

Electrooculographic (EOG) and electromyographic (EMG) signals are among the most common sources of noise in the EEG, as mentioned previously. EOG originates from eye movements, such as blinking and rolling, whereas EMG originates from movements of the surrounding muscles. These artefacts are highly prevalent because it is next to impossible to prevent blinking of the eye and twitching of surrounding muscle groups. As a result of these artefacts ubiquity in EEG signals, it becomes apparentthat there is a need to estimate these noisy signals accurately and remove them to obtain a high-resolution EEG signal upon which fast and accurate diagnosis can be performed.

There have been several methods used in the past to denoise EEG signals. For example, Salis et al. (2013) implement a comparative study of Empirical Mode Decomposition (EMD), Discrete Wavelet Transform (DWT) and Kalman Filter (KF) in an attempt to remove EOG artefacts with different amplitudes from EEG. However, more recent developments have focused on deep-learning to denoise EEG signals, such as Zhang et al. (2021b), who denoise EOG and EMG artefacts using a novel deep-learning-based architecture.

In this work, we present our EEG denoising pipeline based on our Generative Adversarial Network (GAN). We use two datasets to demonstrate the capability of our system. One dataset consists of EEG signals collected using the ANT Neuro eego sports. The other EEG data is the benchmark dataset EEGdenoiseNet presented in Zhang et al. (2021b). We show both the power spectral density (PSD), signal-to-noise ratio (SNR) along with other classical time series similarity measures for quantitative metrics and compare our framework to the benchmark in the literature.

We demonstrate our competitive deep learning technique capable of denoising common artefacts induced in EEG data. Through SNR and other signal evaluation measures, we show the GAN is capable of high-quality denoising that outperforms the current deep learning benchmarks. The following experiments illustrate the potential for use in the brain-computer-interface (BCI) setting.

## 2. Related Work

GANs were initially developed for image generation and improved image synthesis. Although this has gained a lot of traction over recent years, there has been a movement towards implementing GANs for sequence and time series generation, imputation and augmentation; Brophy et al. (2021). In this work, we employ GANs to denoise common EEG artefacts experienced in BCIs.

GANs have been used for EEG generation, and augmentation, as in Palazzo et al. (2017), Hartmann et al. (2018), Corley and Huang (2018), Luo and Lu (2018), Fahimi et al. (2019), and Fahimi et al. (2021). However, few works have explored GANs for denoising time series, particularly where EEG data is concerned.

Gandhi et al. (2018) designed Asymmetric-GANs for denoising EEG time series data. Their model for denoising time series is trained using unpaired training corpora and does not need information about the noise source. Sumiya et al. (2019) denoise mice EEG using adversarial training. Their training process requires a set of noisy signals and clear signals. Although these methods reduce the noise present in the EEG signals, they do not provide specific artefact removal nor solid quantitative evidence of the improvement in the SNR. We improve on this by showcasing GANs as a robust artefact removal/denoising tool via the benchmarking experiments and demonstrate both strong qualitative and, more importantly, quantitative evidence that our GAN is a competitive performer in improving the state-of-the-art denoising methods for EEG artefacts.

Other deep learning methods such as Convolutional Neural Networks (CNNs) and Variational Autoencoders (VAEs) have been used in the past to effectively denoise EEG signals (Hwaidi and Chen, 2021; Zhang et al., 2021a). We demonstrate that the GAN developed in this work is competitive with the state-of-the-art deep learning methods. Many methods proposed in the literature deal with only one artefact type with each architecture. Our model is generalisable to each of the three artefacts explored in this paper; in other words, the same architecture can be retrained to remove more than one artefact type effectively.

## 3. Generative Adversarial Networks

**Figure 2:**
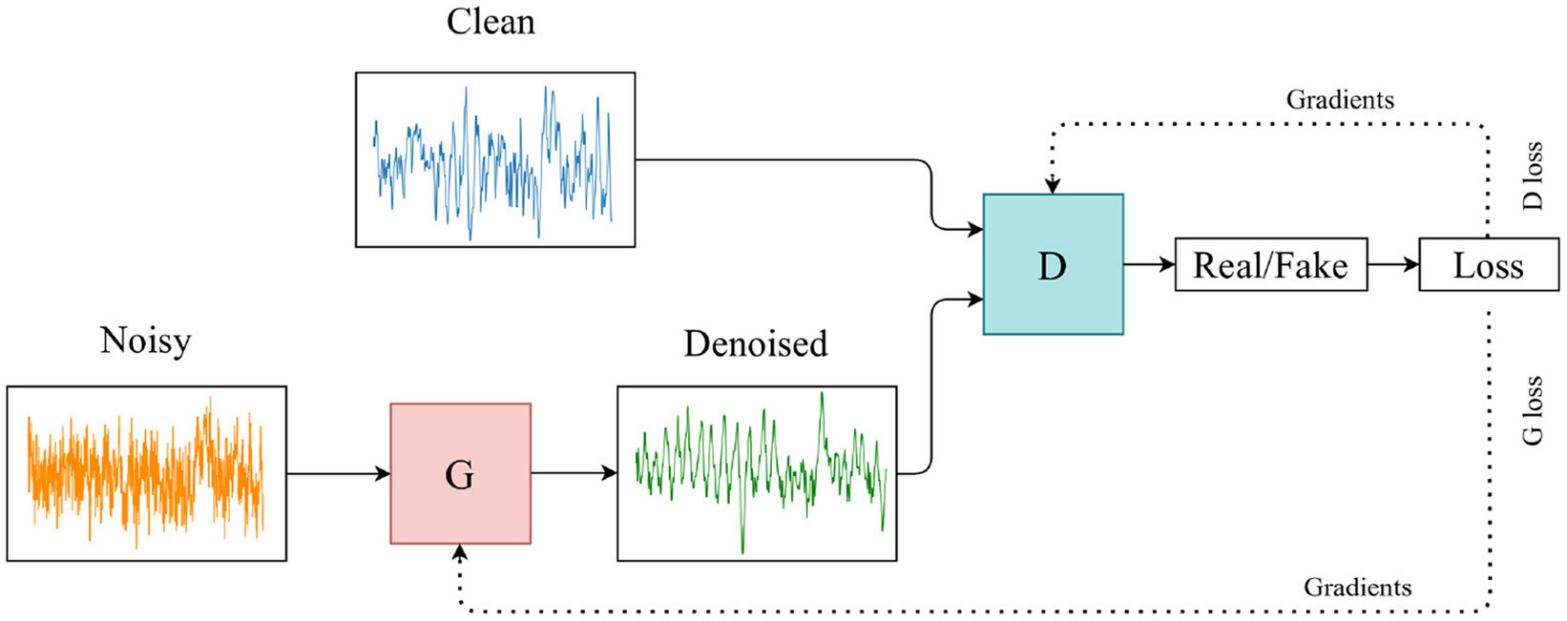
Architecture of the GAN. Noisy EEG to clean EEG.

GANs belong to the family of generative models and are an alternative method of generating synthetic data that do not require domain expertise. They were conceived in the paper by Goodfellow et al. (2014), where a multi-layer perceptron was used for both the discriminator and the generator. The discriminator and generator are typically two neural networks (NN) that are locked in a mini-max game defined by the objective function in Equation (1) where the generator attempts to maximise the failure rate of the discriminator, and the discriminator aims to identify real samples from generated samples, see Figure 2. GANs are most typically used for generating previously unseen data, whether to augment existing datasets or to preserve the privacy of the training data.


(1) $$\begin{array}{l} \underset{G}{min}\;\underset{D}{max}\;V\left(G,D\right)={𝔼}_{x\sim p_{data}\left(x\right)}\left[logD\left(\text{x}\right)\right]\\ \;+{𝔼}_{z\sim p_{\text{z}}\left(z\right)}\left[log\left(1-D\left(G\left(\text{z}\right)\right)\right)\right] \end{array}$$

## 4. Materials and Methods

We implement our GAN that has demonstrated its ability to map from noisy to clean time series modalities. In this work, we use the model as mentioned earlier to learn the noise mapping between our signal pairs and generate clean, denoised EEG data from a noisy EEG signal. Rather than sampling from a latent space for the generator, as is common practice with GANs, we sample the input to the generator from the noisy EEG training data and compare it to the corresponding clean EEG signal in the discriminator. Furthermore, we train this model to denoise a noisy EEG signal segment to its clean or noise-reduced EEG version, which can then be used for further testing and analysis in the BCI environment. Details of our method can be found in the section that follows. See Figure 2 for the architecture of our model.

### 4.1. Computing Platform

The experiments for this project were run on an Nvidia Titan Xp with PyTorch and Google Colaboratory to make the project readily deployable. The experiments are available online^1^.

### 4.2. Datasets

Two open-source datasets were used in this experiment. The first dataset of EEG signals was downloaded from PhysioNet (Schalk et al., 2004; Goldberger et al., 2000). For this dataset, subjects performed different motor/imagery tasks while 64-channel EEG was recorded using the BCI2000 system^2^ and sampled at 160 Hz. Each subject performed 14 experimental runs: two 1-min baseline runs (one with eyes open, one with eyes closed) and three 2-min runs of four motor movement and imagery tasks. We used the baseline eyes open recordings only from this dataset and artificially added mains noise at 50 Hz. The data preprocessing steps for this toy experiment are described in further detail in section 4.4. We refer to this dataset as EEG-50 for the remainder of this work.

**Figure 3:**
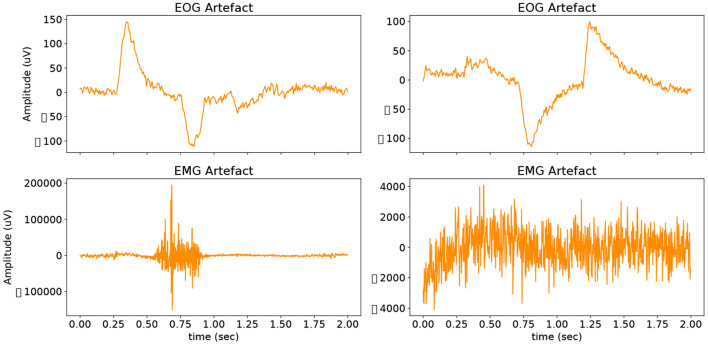
Examples of both EOG and EMG artefacts that have been artificially added to the dataset.

Our second dataset used is the EEGdenoiseNet, a benchmark EEG dataset designed to be implemented with deep learning-based denoising technologies. We use this dataset to act as a performance comparison of our GAN to the models tested in the data collection paper, and we further benchmark our GAN model against other deep-learning EEG denoising architectures previously used in the literature. EEGdenoiseNet contains a total of 13,512 physiological signal segments. Of that, 4,514 records are clean EEG, 3,400 are ocular artefact records, and 5,598 are muscular artefact records. This allows the dataset user to synthesise artificial EOG and EMG artefacts into the clean EEG records, resulting in contaminated EEG segments with the ground truth clean EEG. The EOG data was sampled at 256 Hz, and the EMG data was sampled at 512 Hz. The data preprocessing steps are described in detail in section 4.4. Examples of both EOG and EMG artefacts can be seen in Figure 3.

### 4.3. Model

**Figure 4:**
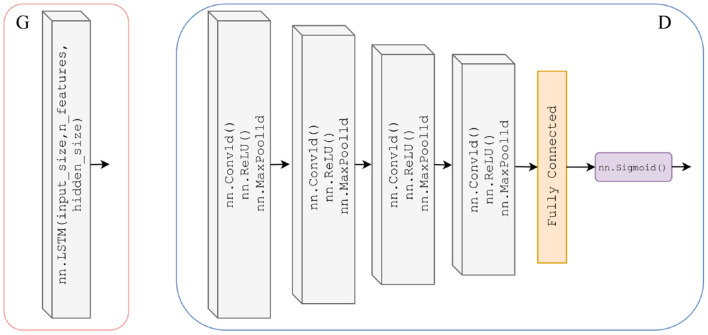
Detailed architecture of generator **(left)** which is a two-layer stacked LSTM with 50 hidden units in each layer and a fully connected layer at the output, with an input size varied to match the segment length for the chosen dataset. Architecture of discriminator **(right)** which is a 4-layer 1-dimensional CNN (ReLU activation and max pooling functions) with a fully connected layer and sigmoid activation function at the output.

The GAN model maps from a noisy time series to a denoised time series, and in this use case, we use it to learn the noise model of the artefact and denoise the EEG signal. We define the generators and discriminators of our GAN as follows. The generator is a two-layer stacked long short-term memory network (LSTM) with 50 hidden units in each layer and a fully connected layer at the output, see Figure 4. The input size is 640 sample points for the EEG-50, 512 sample points for the EEG-EOG and 1,024 sample points for the EEG-EMG datasets. The discriminator is a 4-layer 1-dimensional CNN with a fully connected layer and sigmoid activation function at the output, see Figure 4.

### 4.4. Training

#### 4.4.1. Toy Data Processing

This dataset uses only the resting, eyes open EEG records from the eegmmidb database available on PhysioNet. The dataset is digitised initially at 160 Hz. We segment each EEG record into 4 s long intervals with an overlapping sliding window of 2 s. This yields *12200* EEG records which then have a noisy sinewave of varying amplitudes centred around 50 Hz added to the clean EEG signal. As a result of this, we then have the corresponding clean and noisy signal pairs. The dataset is then normalised before training.

#### 4.4.2. EEGdenoiseNet Data Processing

The EEGdenoiseNet datasets use the same training setup as described in the original paper. First, the noisy EEG segments are created by linearly mixing the clean EEG segments with the EOG and EMG artefacts according to Equation (2):


(2) $$\begin{array}{l} y=x+\lambda \cdot n \end{array}$$

where *x* is the clean EEG signals, *n* is the artefact (either EOG or EMG), and λ is a hyperparameter that controls the SNR levels of the noisy EEG signal *y*.

The contaminated signals are from a combination of EEG segments and ocular or myogenic artefact segments, with 80% for generating the training set and 20% for generating the test set. Each set was generated by randomly linearly mixing EEG segments and EMG or EOG artefact segments according (Equation 2), with SNR ranging from ten different SNR levels (–14, –12, –10, –8, –6, –4, –2, 0, 2, and 4dB) rather than the 10 levels of (–7, –6, –5, –4, –3, –2, –1, 0, 1, and 2dB) in Zhang et al. (2021b). This procedure expanded each dataset to ten times its original size. The clean EEG records act as ground truth, and the corresponding mixed records are the noisy EEG.

#### 4.4.3. Objective Function

The loss function of our GAN framework is calculated as in Equations (3) and (4). Here, a is the label for the generated samples, b is the label for the real samples, and c is the hyperparameter that G wants D to recognise the generated samples as real samples.


(3) $$\begin{array}{l} \underset{D}{min}\;V_{LSGAN}\left(D\right)=1/2*{𝔼}_{x\sim p_{data}\left(x\right)}\left[{\left(D\left(\text{x}\right)-b\right)}^{2}\right]\\ \;+1/2*{𝔼}_{z\sim p_{\text{z}}\left(z\right)}\left[{\left(D\left(G\left(\text{z}\right)\right)-a\right)}^{2}\right] \end{array}$$

(4) $$\begin{array}{l} \underset{G}{min}\;V_{LSGAN}\left(G\right)=1/2*{𝔼}_{z\sim p_{\text{z}}\left(z\right)}\left[{\left(D\left(G\left(\text{z}\right)\right)-c\right)}^{2}\right] \end{array}$$

### 4.5. Evaluation

To quantitatively evaluate our denoised data, we look at the SNR vs. relative root mean squared error (RRMSE), Pearson's correlation coefficient (CC) and the power ratios of the associated EEG bands across the signals. We also qualitatively evaluate our results through a visual inspection in both the time series domain and the frequency domain via the PSD of the EEG.

We use SNR to compare the level of the desired EEG signal to the level of noise/artefact present in the signal. The formula for SNR is given as in Equation (5), again, where *x* is the EEG signal of interest, *n* is the artefact, and λ is the hyperparameter that controls the SNR.


(5) $$\begin{array}{l} SNR=10*log_{10}\frac{RMS\left(x\right)}{RMS\left(\lambda \cdot n\right)} \end{array}$$

The Root Mean Square (RMS) of a signal is given in Equation (6). N is defined as the number of samples in the EEG signal segment *a*, and *a*_*i*_ denotes the *i*_*th*_ sample in the EEG signal. *N* = 512 and 1, 024 for the EOG and EMG signals, respectively.


(6) $$\begin{array}{l} RMS\left(x\right)=\sqrt{\frac{1}{N}\overset{N}{\underset{i=1}{\sum }}a_{i}^{2}} \end{array}$$

RRMSE is given in Equation (7) for the temporal/time domain and in Equation (8) for the frequency/spectral domain. *f*(*y*) is the noisy signal passed through our model; in our case, it becomes the denoised signal generated by the GAN. We calculate the PSD using the FFT-length equal to the total length of the EEG input segment with a Hanning window.


(7) $$\begin{array}{l} RRMSE_{temporal}=\frac{RMS\left(f\left(y\right)-x\right)}{RMS\left(x\right)} \end{array}$$

(8) $$\begin{array}{l} RRMSE_{spectral}=\frac{RMS\left(PSD\left(f\left(y\right)\right)-PSD\left(x\right)\right)}{RMS\left(PSD\left(x\right)\right)} \end{array}$$

Pearson's correlation coefficient is shown in Equation (9), where *Cov* is the covariance and *Var* is the variance of the signals *f*(*y*) and *x*.


(9) $$\begin{array}{l} CC=\frac{Cov\left(f\left(y\right),x\right)}{\sqrt{Var\left(f\left(y\right)\right)Var\left(x\right)}} \end{array}$$

## 5. Results

### 5.1. EEGdenoiseNet Experiment

**Figure 5:**
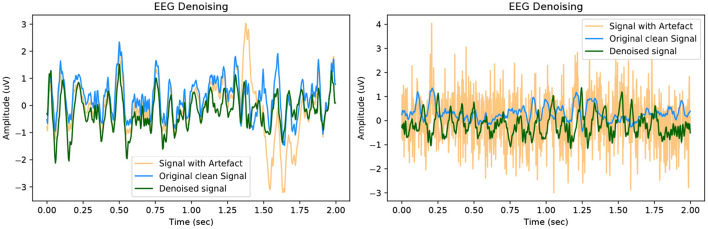
Example of denoised time series EEG corrupted with **(left)** EOG artefact and **(right)** EMG artefact. The signals contain an artificial offset for visualisation purposes.

In this section, we showcase our model's performance on the EEGdenoiseNet dataset. We present both quantitative and qualitative evidence of our methods competitive performance against the benchmark established in the original paper. A qualitative example of high-fidelity denoised EEG for our GAN model is presented in Figure 5. For visualisation purposes, an offset is artificially introduced to the ground truth and denoised EEG signals. Further examples of denoised EEG with the corresponding noisy EEG and ground truth can be found in the Supplementary Material.

**Figure 6:**
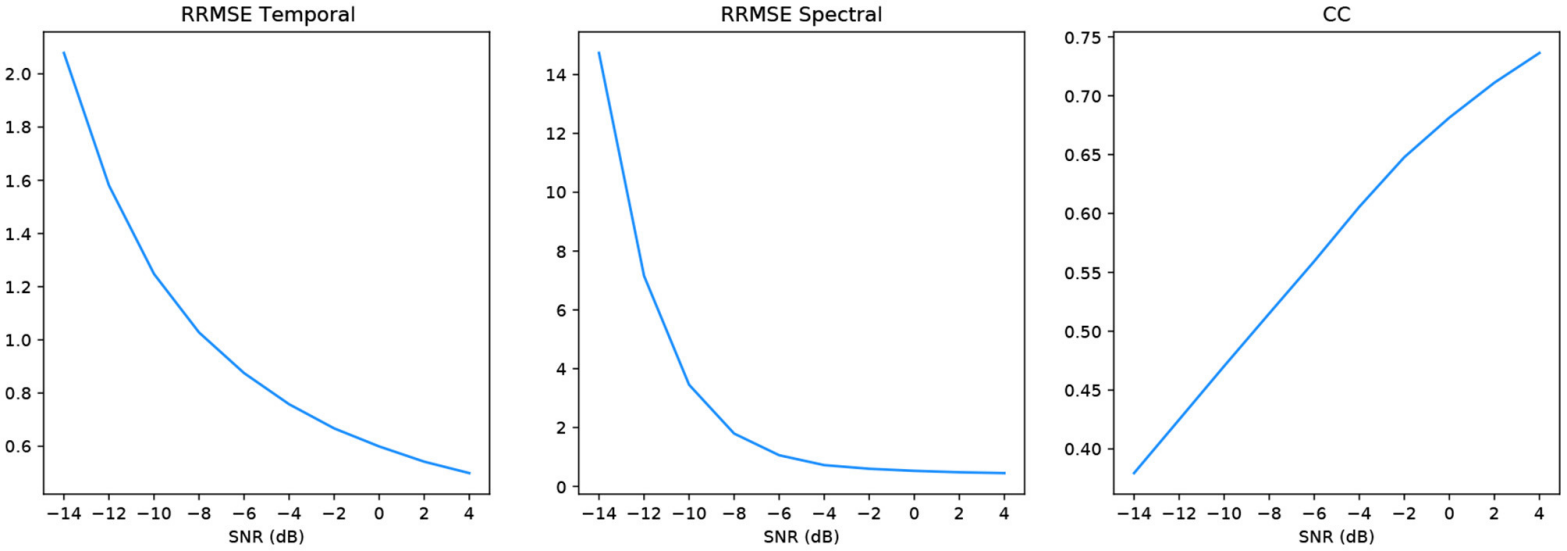
Metrics of the EEG-EOG signals shown in Figure 5 (left).

**Figure 7:**
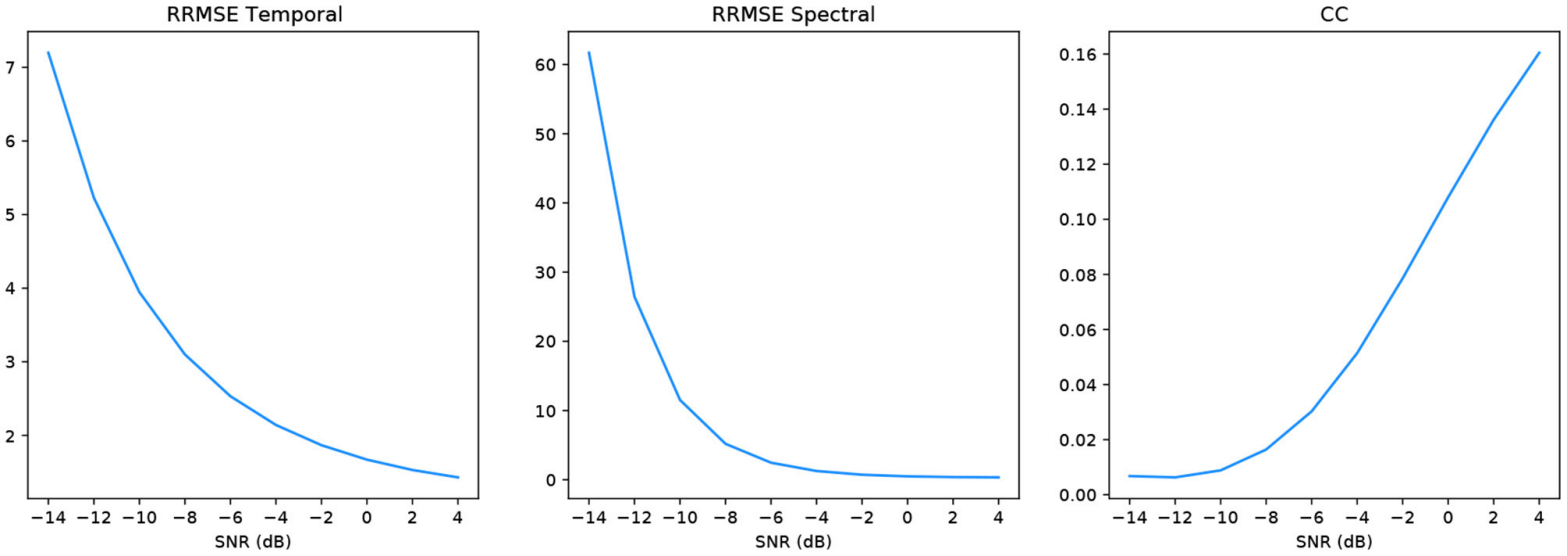
Metrics of the EEG-EMG signals shown in Figure 5 (right).

In keeping with the benchmark evaluation metrics we present the *RRMSE*_*temporal*_, *RRMSE*_*spectral*_ and *CC* graphs at all our SNR levels. It should be noted that the performance of our model outperforms the other models in the benchmark experiment, we also provide results from deep learning models that have been implemented in the literature as a comparison to our GAN. For all SNR levels, our GAN performs extremely well, see section 5.1.1 for further details. The graphs in Figure 6 correspond to the denoised EEG signal in Figure 5 (left). Similarly, the graphs in Figure 7 correspond to the denoised EEG signal in Figure 5 (right). For both EOG and EMG our model outperforms the benchmarks across *RRMSE*_*temporal*_, *RRMSE*_*spectral*_ and *CC*. In general, the denoising capability of our model improves as the SNR improves. The CC for the EEG-EMG experiment does increase as the SNR improves, however, this is one of the few metrics that needs further experimentation on to improve.

**Figure 8:**
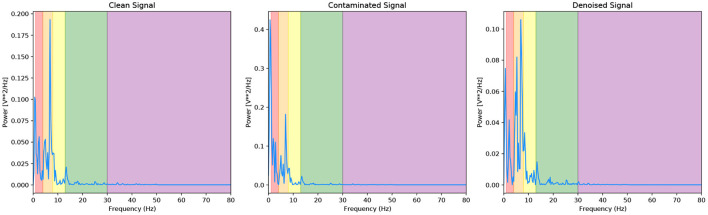
PSD of the EEG-EOG signals shown in Figure 5 (left) with corresponding EEG bands.

We present a final metric to evaluate our denoising model quantitatively, and it is the power present in the different EEG bands. Figure 8 and Table 1 are the corresponding PSD and power band ratios for the EEG signals shown in Figure 5 (left), respectively. It can be seen that the high power low-frequency components in the delta band are present in the EOG contaminated signal are removed from the denoised signal. We present results for the noisy EEG at −14*dB* as this can be considered the worst-case scenario for the denoising GAN model. As can be observed, the model effectively removes the EOG artefacts in the contaminated data.

**Table 1:** Power ratios of different frequency bands before and after EOG artefact removal.

**Denoising method**	**Delta**	**Theta**	**Alpha**	**Beta**	**Gamma**
GAN (-14dB)	0.3020	0.4091	0.1647	0.1023	0.0217
Ground truth	0.2769	0.4299	0.1349	0.1158	0.0424
Contaminated signal (-14dB)	0.7999	0.1280	0.0319	0.0289	0.0113

**Figure 9:**
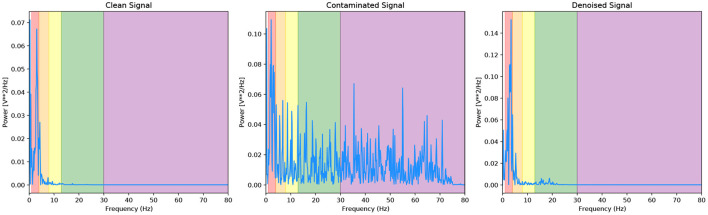
PSD of the EEG-EMG signals shown in Figure 5 (right) with corresponding EEG bands.

Likewise, Figure 9 and Table 2 are the PSD and power band ratios that corresponds to the EEG signals in Figure 5 (right). Again, it is apparent that the high-frequency noise in the beta and gamma bands present in the EMG contaminated EEG is suppressed in the denoised signal. For both EOG and EMG datasets, the power across the denoised EEG frequency bands is recovered in the denoised signal.

**Table 2:** Power ratios of different frequency bands before and after EMG artefact removal.

**Denoising method**	**Delta**	**Theta**	**Alpha**	**Beta**	**Gamma**
GAN (–14dB)	0.6528	0.2243	0.0299	0.0908	0.0023
Ground truth	0.6458	0.2213	0.0658	0.0671	1.19e-10
Contaminated signal (–14dB)	0.0612	0.0471	0.0796	0.2981	0.5140

#### 5.1.1. Benchmarking Deep Learning Models

It is important to frame our model in the context of other deep learning frameworks, and as such, we benchmark our model against neural networks that have been used successfully in the past to denoise EEG data. We implemented various CNNs, VAEs and Convolutional Autoencoders (CAEs) with the same training process as the GAN and 5-fold cross-validation. As GANs are not intuitive, the training and validation losses can be misleading. Still, it is worth monitoring the losses for convergence between the discriminator and generator as the original concept is a zero-sum game between the two NNs. Rather than observing a validation loss, it is better practice to quantitatively and qualitatively evaluate the data generated by the GAN.

We compare each NN models' denoised signals to the ground truth. The comparison we present is the ability of each model to preserve the power ratios across the various EEG frequency bands. We compute the cosine similarity of the power ratio across the frequency bands between the denoised and ground truth at the -14dB level, as can be seen in Table 3.

**Table 3:** Co-sine similarity score of the different frequency bands after artifact removal (to ground truth).

**Denoising model**	**EOG-Score**	**EMG-score**
GAN	0.995	0.998
SimpleCNN	0.985	0.9766
C-VAE	0.982	0.9916
CAE	0.819	0.9202
Novel-CNN	0.793	0.9914

Ranking the DL-models in terms of the EEG frequency preservation across bands shows that the GAN outperforms the other models.

To truly demonstrate the usefulness of deep learning models, it should be shown that the denoising method can improve downstream tasks. However, we cannot readily apply this dataset to a classification task with this dataset. Instead, to demonstrate the effectiveness of the denoised data, we trained a classifier to distinguish between the original ground truth data and noisy data. Following this, we test the trained classifier on the ground truth vs. noisy data and then again on the denoised data vs noisy data. Finally, we compare the F1-score of both classifiers. The F1-score of the original ground truth data is 0.8987, with an accuracy of 88.75%. Whereas, when using the denoised data, the F1-score reduces to 0.7799 and accuracy of 77.94%.

### 5.2. Toy Experiment

**Figure 10:**
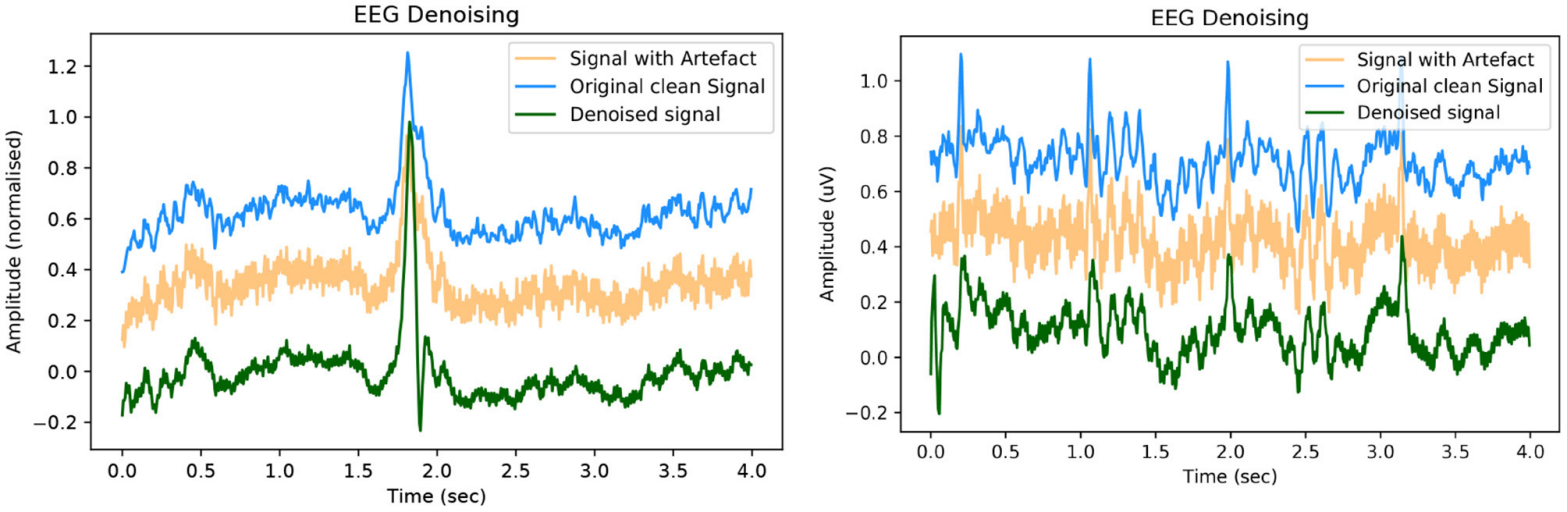
Example of denoised time series EEG corrupted with 50 Hz mains noise. The signals contain an artificial offset for visualisation purposes. Note the appearance of an ocular artefact in both examples.

Here we present brief examples of the GAN's performance on the toy EEG-50 dataset. Further examples of the results from this experiment can be found in the Supplementary Material. Examples of the denoised time series EEG signal can be seen in Figure 10.

Similar to the EEGdenoiseNet example, we demonstrate the performance of our model at removing the 50 Hz noise through the use of metrics. As this dataset was not divided into SNR levels we return one set of values for the metrics and they are as follows; *RRMSE*_*t*_*emporal* = 0.05, *RRMSE*_*s*_*pectral* = 0.1 and *CC* = 0.89. These metrics show that our model is more than capable of learning the noise model between our signal pairs.

**Figure 11:**
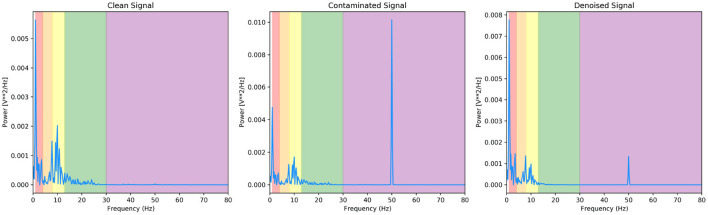
PSD of the EEG-50 signals shown in Figure 10 (right) with corresponding EEG bands.

Again, to quantitatively evaluate our denoising model, we illustrate the power present in the different EEG bands of our signals. Figure 11 and Table 4 are the corresponding PSD and power band ratios for the EEG signals shown in Figure 10 (left), respectively. It can be seen that the high power high-frequency components, centred around 50 Hz in the gamma band, is present in the contaminated signal and is heavily suppressed in the denoised signal. Once again, the model effectively reduces the mains noise artefacts in the contaminated data.

**Table 4:** Power ratios of different frequency bands before and after 50 Hz noise removal.

**Denoising method**	**Delta**	**Theta**	**Alpha**	**Beta**	**Gamma**
GAN	0.6528	0.2243	0.0299	0.0908	0.0023
Ground truth	0.6046	0.2212	0.0658	0.0671	1.19e-10
Contaminated signal	0.0612	0.0472	0.0796	0.2981	0.5140

## 6. Discussion and Conclusion

In this work, we have introduced a novel deep learning framework capable of denoising and evaluating EEG data. In addition, we have presented our qualitative and quantitative analysis that demonstrated that our model outperforms the benchmarks on many of the metrics provided in the original paper. Thus, we build on and contribute to the initial experiments and show that our model is currently state-of-the-art in this deep learning-based EEG denoising experiment.

**Figure 12:**
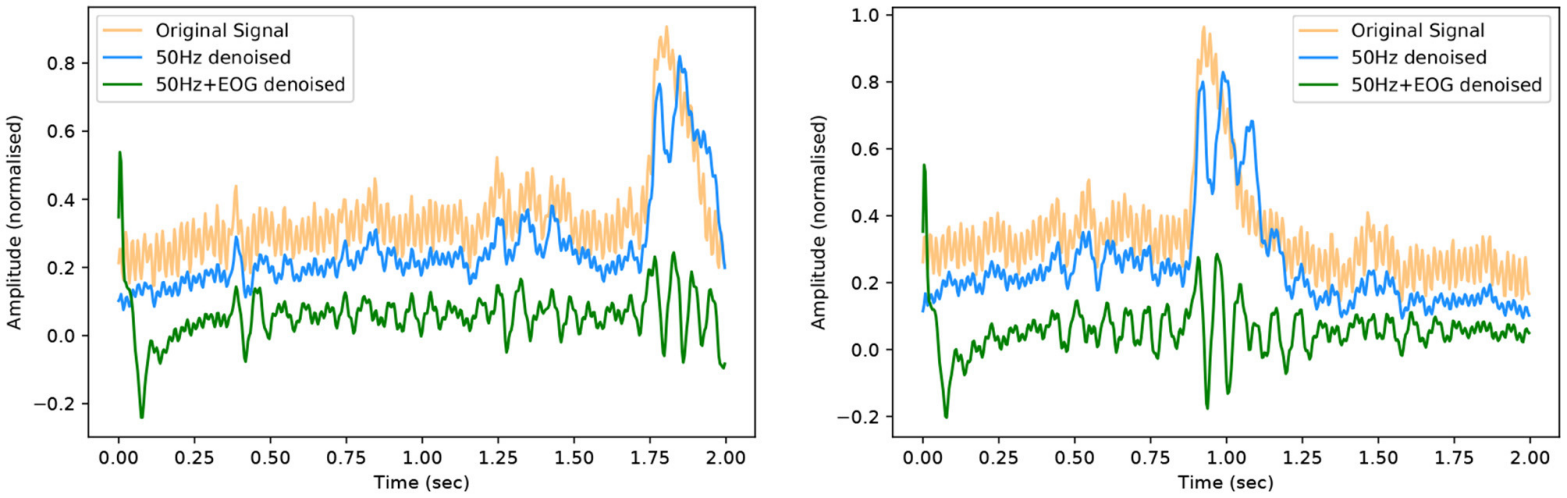
Denoised EEG signals following 50 Hz and EOG artefact removals.

To demonstrate the full capability of these models and that they have not overfitted to their respective datasets, we pass a signal with both 50 Hz noise and EOG artefact through our models. This signal is taken from the eegmidb dataset that has a natural ocular artefact introduced from the subject. We then artificially introduce 50 Hz noise to the signal. This corrupted EEG signal is then denoised using the EEG-50 model, the output of which is then resampled and passed through the EEG-EOG model. Two examples of EEG signals at each stage of the denoising process are shown below in Figure 12.

GANs can be implemented as supervised and unsupervised learning methods making them ideal for portable physiological monitoring systems. Furthermore, with the integration of AI systems into wearable technologies, our framework lays the foundation for continuous, portable and remote EEG and BCI devices with less noisy and more stable brain signals. Using these methods to produce high fidelity and reliable EEG data may be a solution for clinicians to remotely and accurately monitor patients' brain activities states.

## Data Availability Statement

The original contributions presented in the study are included in the article/Supplementary Material, further inquiries can be directed to the corresponding author.

## Author Contributions

EB: conceptualisation, methodology, software, and validation. EB and TW: writing—original draft preparation. EB, PR, AF, MD, GB, and TW: writing—review and editing. TW: supervision. All authors have read and agreed to the published version of the manuscript.

## Funding

This work is funded by Science Foundation Ireland under grant numbers 17/RC-PhD/3482 and SFI/12/RC/2289\P2 and by the Flemish Government (AI Research Program).

## Conflict of Interest

The authors declare that the research was conducted in the absence of any commercial or financial relationships that could be construed as a potential conflict of interest.

## Publisher's Note

All claims expressed in this article are solely those of the authors and do not necessarily represent those of their affiliated organizations, or those of the publisher, the editors and the reviewers. Any product that may be evaluated in this article, or claim that may be made by its manufacturer, is not guaranteed or endorsed by the publisher.

## Footnotes

^1^GitHub Repository: https://github.com/Brophy-E/DenoiseEEG-GAN

^2^ http://www.bci2000.org
