# Supplementary material for: Denoising EEG Signals for Real-World BCI Applications Using GANs
Source: Front Neuroergon. 2022 Jan 13;2:805573. doi: 10.3389/fnrgo.2021.805573 (PMC10790876; doi:10.3389/fnrgo.2021.805573)
Supplement: Supplementary file 1 [file Data_Sheet_1.PDF]

Supplementary Material

1 SUPPLEMENTARY TABLES AND FIGURES

1.1 Figures

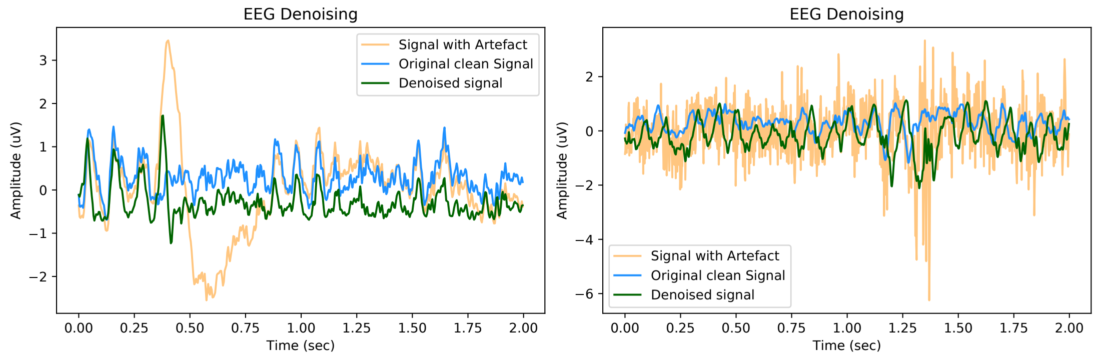

Figure S1. Example of denoised time-series EEG corrupted with (left) EOG artefact and (right) EMG artefact. The signals contain an artificial offset for visualisation purposes.

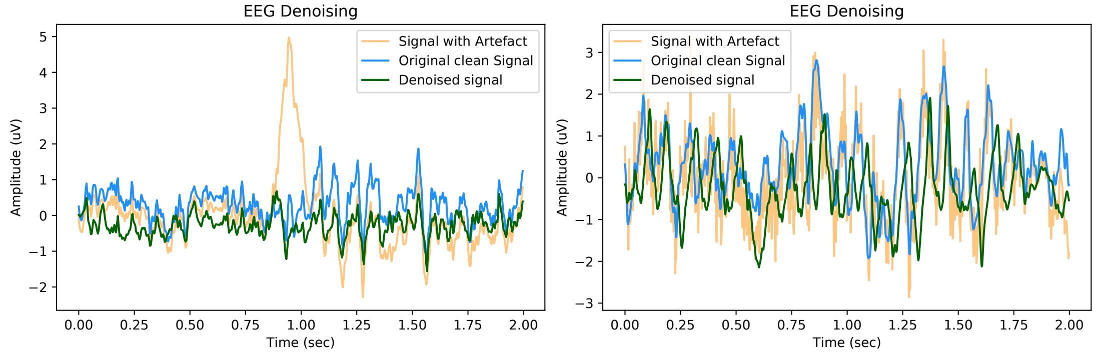

Figure S2. Example of denoised time-series EEG corrupted with (left) EOG artefact and (right) EMG artefact. The signals contain an artificial offset for visualisation purposes.

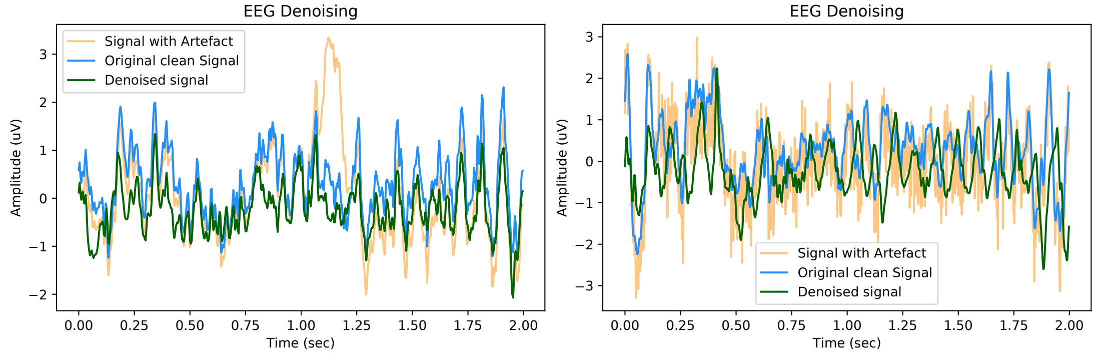

Figure S3. Example of denoised time-series EEG corrupted with (left) EOG artefact and (right) EMG artefact. The signals contain an artificial offset for visualisation purposes.

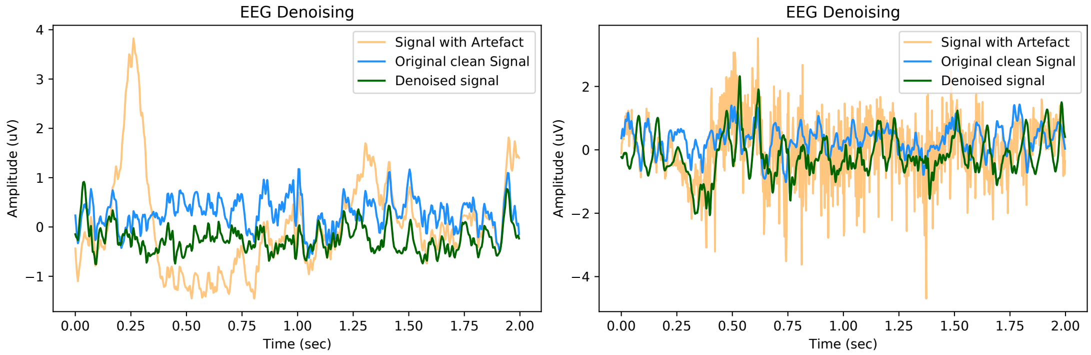

Figure S4. Example of denoised time-series EEG corrupted with (left) EOG artefact and (right) EMG artefact. The signals contain an artificial offset for visualisation purposes.

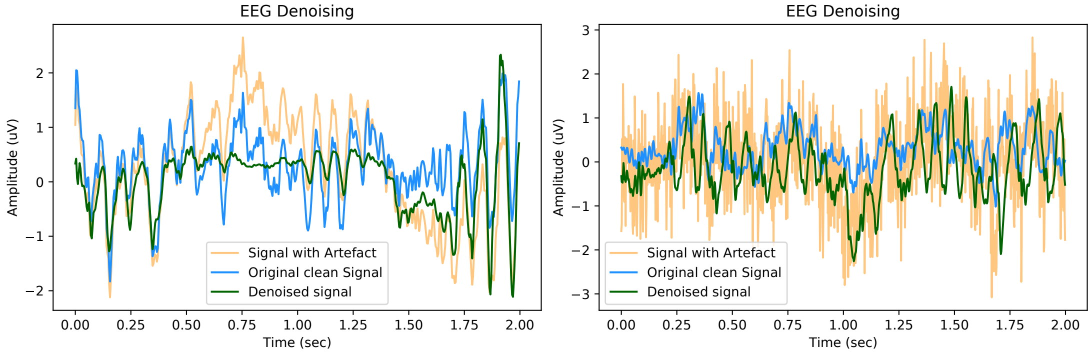

Figure S5. Example of denoised time-series EEG corrupted with (left) EOG artefact and (right) EMG artefact. The signals contain an artificial offset for visualisation purposes.

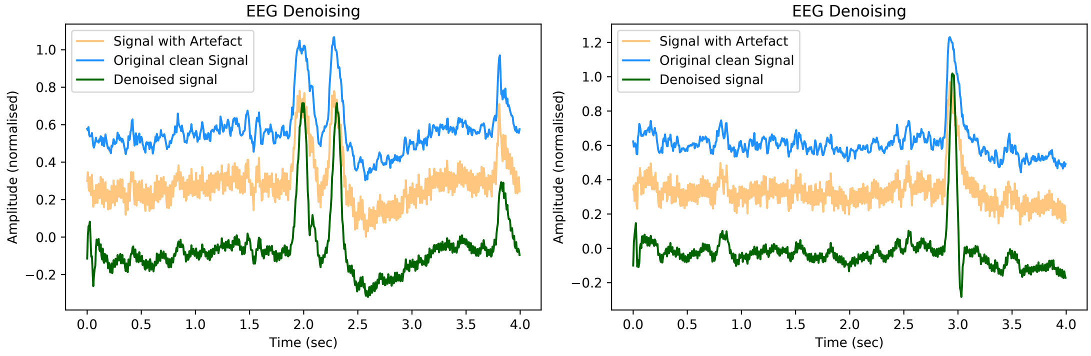

Figure S6. Example of denoised time-series EEG corrupted with 50 Hz mains noise. The signals contain an artificial offset for visualisation purposes.

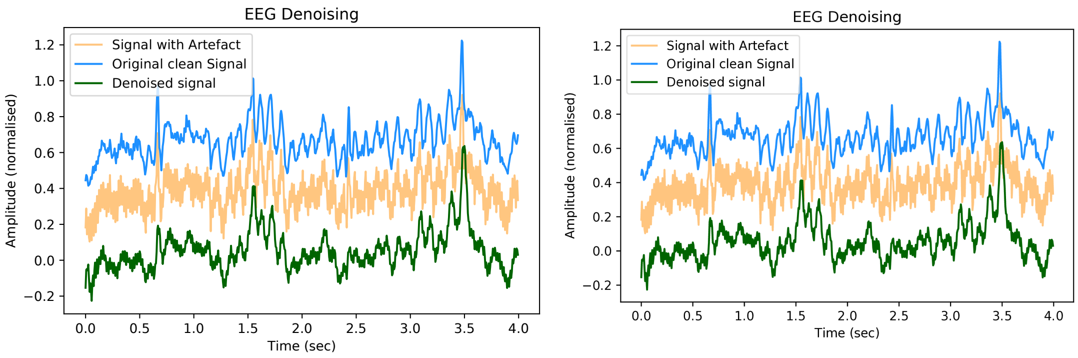

Figure S7. Example of denoised time-series EEG corrupted with 50 Hz mains noise. The signals contain an artificial offset for visualisation purposes.
